# A Comprehensive Guide to Enzyme Immobilization: All You Need to Know

**DOI:** 10.3390/molecules30040939

**Published:** 2025-02-18

**Authors:** Marina Simona Robescu, Teodora Bavaro

**Affiliations:** Department of Drug Sciences, University of Pavia, Viale Taramelli 12, I-27100 Pavia, Italy

**Keywords:** enzyme immobilization, immobilization techniques, enzyme engineering, biocatalysis, sustainable processes

## Abstract

Enzyme immobilization plays a critical role in enhancing the efficiency and sustainability of biocatalysis, addressing key challenges such as limited enzyme stability, short shelf life, and difficulties in recovery and recycling, which are pivotal for green chemistry and industrial applications. Classical approaches, including adsorption, entrapment, encapsulation, and covalent bonding, as well as advanced site-specific methods that integrate enzyme engineering and bio-orthogonal chemistry, were discussed. These techniques enable precise control over enzyme orientation and interaction with carriers, optimizing catalytic activity and reusability. Key findings highlight the impact of immobilization on improving enzyme performance under various operational conditions and its role in reducing process costs through enhanced stability and recyclability. The review presents numerous practical applications of immobilized enzymes, including their use in the pharmaceutical industry for drug synthesis, in the food sector for dairy processing, and in environmental biotechnology for wastewater treatment and dye degradation. Despite the significant advantages, challenges such as activity loss due to conformational changes and mass transfer limitations remain, necessitating tailored immobilization protocols for specific applications. The integration of immobilization with modern biotechnological advancements, such as site-directed mutagenesis and recombinant DNA technology, offers a promising pathway for developing robust, efficient, and sustainable biocatalytic systems. This comprehensive guide aims to support researchers and industries in selecting and optimizing immobilization techniques for diverse applications in pharmaceuticals, food processing, and fine chemicals.

## 1. Introduction

In the pursuit of sustainable solutions, biocatalysis has emerged as a central strategy promoted by the chemical industry to align with green chemistry principles. The ability of biocatalysts to facilitate reactions under mild conditions while offering high selectivity positions them as a promising alternative for sustainable industrial applications [1,2].

Consequently, the use of biocatalysis is expanding across various sectors, including fine chemicals, pharmaceuticals, food processing, and chemical manufacturing [3,4,5,6]. However, despite their advantageous properties, the full potential of enzymes for industrial applications remains underutilized. The challenges of enzyme stability under extreme conditions, such as pH levels, high temperatures, and exposure to solvents, surfactants, or metal ions, limit their broader application. Additional challenges include short shelf life and, most critically, difficulties in enzyme recovery and recycling [7,8,9]. To address these limitations, the biocatalyst can be engineered through immobilization techniques. Immobilization has been extensively utilized (i) to improve enzyme stability and reusability, allowing for continuous or repeated batch operations; (ii) to simplify enzyme separation from the product, enhancing compatibility with various processes; and (iii) to reduce the need for extensive downstream processing, making the process cost-effective, reliable, and efficient. The engineering of enzymes through immobilization has, thus, become an essential focus in the biocatalysis field [10]. Indeed, nowadays, enzyme immobilization has evolved into a powerful tool for biocatalyst engineering, complementing strategies such as recombinant DNA technology, protein engineering, high-throughput technology, genomics, and proteomics [11,12]. The combination of recombinant DNA technology and enzyme engineering has greatly advanced the large-scale production of enzymes with desirable properties. Protein engineering techniques, including site-directed mutagenesis and in vitro evolution allow for precise manipulation to achieve attributes like chemoselectivity, regioselectivity, stereoselectivity, long-term stability, activity in high substrate concentrations, and tolerance to organic solvents. However, these approaches are often labor-intensive, costly, and can lack long-term operational stability. Additionally, they can pose challenges for enzyme recovery and reuse. Despite its benefits, enzyme immobilization presents its own challenges, including potential decreases in enzyme activity due to conformational changes or uncontrolled orientation, risks of enzyme denaturation, altered kinetic properties, mass transfer limitations, and reduced catalytic efficiency with insoluble substrates. However, not all immobilization systems are affected by these issues. Robust enzyme applications often benefit from a combined approach, where protein engineering precedes immobilization, maximizing stability and performance. To create robust biocatalysts, protein engineering methods can be applied prior to immobilization to maximize stability and performance across various immobilization systems. Additionally, enzyme engineering techniques can also be applied in order to achieve a more rational immobilization protocol. By introducing into the sequence of the enzyme specific tags or unique (un)natural amino acid residues the orientation of the biocatalyst during immobilization can be controlled allowing to obtain a specific interaction [13].

An effective immobilization system should securely anchor the enzyme to prevent any unintended release that could contaminate products and lead to enzyme loss, thereby reducing catalytic activity. Additionally, immobilization is closely linked to stability, as only sufficiently stable biocatalysts can be reused efficiently [14,15]. However, immobilization alone does not guarantee enzyme stabilization. In cases where poor immobilization protocols allow uncontrolled interactions between enzyme and support, immobilization can actually reduce enzyme stability compared to the free enzyme. Poorly designed immobilization protocols, particularly those that permit uncontrolled interactions between the enzyme and the support, can actually reduce stability compared to soluble enzymes [16].

The immobilization process, defined as the incorporation of an enzyme within or on a porous solid support, requires careful consideration of the functional groups on both the support and enzyme and the immobilization method itself [17]. Proper selection of these components enables optimal immobilization outcomes. It is, however, difficult, if not impossible, to formulate a general immobilization strategy because the method used has to be not only protein- but also application-specific. This topic will be extensively explored in this manuscript, providing a thorough overview of immobilization techniques and considerations for maximizing enzyme performance.

## 2. Classical Non-Specific Immobilization

**Figure 1:**
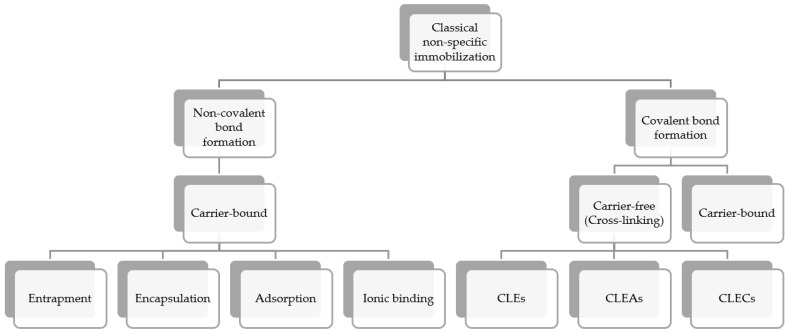
Carrier-bound and carrier-free immobilization methods. Abbreviations: CLEs–cross-linked enzymes; CLECs—cross-linked enzyme crystals; CLEAs—cross-linked enzyme aggregates.

Classical non-specific immobilization protocols rely on the presence of reactive natural amino acids exposed on the enzyme surface that are able to react with an opportunely derivatized carrier (carrier-bound immobilization techniques) or with other enzyme molecules (carrier-free immobilization techniques) (Figure 1). Classical non-specific immobilization technique does not allow a fine control of the orientation of the enzyme during immobilization, even if when an enzyme is immobilized by using a specific immobilization protocol, the enzyme can interact with some exposed regions and not with others, suggesting that all of the immobilized protein molecules have a specific inaccessible surface area [13]. However, the immobilization of an enzyme involving different areas of the enzyme may affect all of its properties (from activity to selectivity or stability). Conventional enzyme immobilization techniques include both carrier-bound and carrier-free methods (Figure 1) [7,18,19]. Consequently, these methods may involve the formation of a strong chemical bond (covalent interaction), weak interactions (adsorption and ionic interactions), or including the absence of any interaction between the protein and the support (cross-linking).

### 2.1. Classical Non-Covalent Immobilization

Classical non-covalent strategies for enzyme immobilization on solid supports typically rely on physical or electrostatic interactions—such as hydrophobic, van der Waals, hydrogen bonds, or ionic forces—between functional groups on the enzyme surface and the support. The main drawback is the undesired release of the enzyme during the reaction course, even when multiple weak physical interactions are used (e.g., adsorption or ion exchange). Indeed, these approaches generally involve an overlap of several weak forces, resulting in non-specific binding. However, they are the simplest and least expensive methods, and they do not significantly alter the enzyme conformation, thereby preserving its catalytic activity [20].

Among the classical strategies for non-covalent enzyme immobilization on carriers, immobilization by metal affinity is also noteworthy. This approach requires recombinant production of the protein with a six- to ten-histidine sequence (known as a His-tag) attached to its *N*- or *C*-terminus. In this review, this method is further discussed in Section 3.1.1.

#### 2.1.1. Entrapment

Entrapment is an immobilization technique that involves enclosing enzymes or cells within a fiber network. It can also be described as the encapsulation of enzymes within a support material, either with a lattice structure or integrated into polymer membranes. By carefully adjusting the pore size of the polymeric network, it is possible to prevent enzyme leakage while allowing the free diffusion of substrates and products [21].

Isolated enzymes have been immobilized using this technique. Alkaline protease was entrapped in mesoporous silica and zeolite (immobilization yield 63.5% and 79.77%, respectively) and utilized as a milk coagulant in the production of dairy products, while laccase was immobilized on alginate beads for dye removal from water [21,22,23].

One of the main advantages of this technique is that enzymes do not chemically interact with the polymers, which significantly reduces the risk of denaturation. Additionally, entrapment enables high enzyme loading capacity, enhances mechanical stability, and is relatively inexpensive to implement. The material used for entrapment can also be tailored to optimize the enzyme microenvironment by adjusting properties such as pH, polarity, or amphiphilicity. Despite its benefits, entrapment has certain limitations. As polymerization progresses, the resulting increase in matrix thickness can lead to mass transfer resistance, making it difficult for substrates to reach the enzyme active site. Furthermore, if the pore sizes of the support material are too large, enzymes may leak out. The most widely used method for enzyme entrapment is the gelation of polycationic or polyanionic polymers using multivalent counterions. Other techniques include photopolymerization, sol-gel processes, and electropolymerization [24].

A more recent advancement in enzyme immobilization is the use of membranes as carriers. Enzyme immobilization on membranes (EIM) has emerged as a promising strategy for enhancing enzymatic stability, reusability, and efficiency in biocatalytic applications [25]. In this context, fumarase has been entrapped within polysulfone membranes for the production of l-malic acid, a compound widely used in food and pharmaceutical industries. By confining the enzyme within the membrane’s porous network, this system maintains high enzymatic activity over extended periods, though substrate diffusion may become a limiting factor. Another example is the co-immobilization of glucose oxidase and catalase in polymeric composite membranes, where both enzymes work synergistically for glucose oxidation. This multienzymatic system is particularly valuable in biosensing applications, ensuring selective enzymatic reactions while reducing external interferences [26].

#### 2.1.2. Encapsulation

Encapsulation is similar to entrapment in that both enzymes and cells are suspended in solutions but within a controlled environment. The encapsulation technique is specifically intended for sensitive enzymes and cells, confining them within small vesicles that have porous membranes [27]. Ionotropic gelation of alginates and silica-based nanoporous sol-gel glasses has proven to be an effective method for enzyme encapsulation. Few examples are reported in the literature regarding enzyme immobilization through encapsulation. Notably, Nitto Chemical (now Mitsubishi Rayon, Tokyo, Japan) developed a process in which the conversion of acrylonitrile to acrylamide, catalyzed by bacterial nitrile hydratases, is achieved. This biotransformation is performed using immobilized whole cells encapsulated within a cross-linked gel composed of 10% (*w*/*v*) polyacrylamide and dimethylaminoethylmethacrylate [18].

Recently, horseradish peroxidase encapsulated into tyramine-alginate beads was applied for phenol degradation in the treatment of wastewater. After four cycles, the immobilized horseradish peroxidase preserved >60% activity, with a phenol removal efficiency reported at 96% [28]. α-Lactalbumin nanotubes were used as carriers for lipase immobilization. This derivative released 50% more free fatty acids compared to soluble lipases. In low-fat cheeses, these nanotubes doubled the release of free fatty acids compared to regular cheeses with the same fat content, enhancing the flavor of the low-fat cheese [29]. However, this technique comes with certain constrains. One of the main issues is the diffusion problem, which can be particularly severe and may result in membrane rupture if the reaction products accumulate too quickly [30].

#### 2.1.3. Adsorption

Immobilization by adsorption is the easiest and least invasive reversible immobilization technique. This method provides weak enzyme binding (van der Waals and hydrophobic interactions), and lipases immobilized on hydrophobic resins, especially polymethyl methacrylates, are extensively used. One of the most notable examples of lipase adsorption onto organic resins is the widely utilized *Candida antarctica* lipase B (CaLB), commercially available in its immobilized form as Novozym 435^®^. This derivative features the enzyme adsorbed onto a macroporous resin composed of polymethyl/butyl methacrylate cross-linked with divinylbenzene, and the immobilized lipase activity was preserved after 3 h at 60 °C [31]. Hydrophobic resin-immobilized lipases, including CaLB, are commonly used for the production of various edible oils, such as cocoa butter substitutes, fats for infant formulas, emulsifiers, and omega-3 fish oil derivatives [32,33]. In addition, the use of immobilized lipases as chemo- and regioselective catalysts is especially valuable in organic chemistry for the synthesis of modified carbohydrates for pharmaceutical applications [34,35,36,37,38]. 

Lipases typically have a hydrophobic surface with a lid that shields the active site, likely to prevent the hydrolysis of non-lipid esters within the cell. The enzyme is activated when the lid opens, which occurs in hydrophobic environments such as the surface of a lipid droplet. By using a hydrophobic carrier, the lipase can bind while also keeping the lid locked in its open position.

Other examples of enzymes immobilized by adsorption and applied in the pharmaceutical field involve transaminases, which are often used for the synthesis of chiral molecules. These enzymes, immobilized on hydrophobic resins, enable stereoselective reactions, which are essential for the production of pharmaceutical active ingredients. For instance, immobilized transaminases have been used in the synthesis of drugs for the treatment of diabetes, such as sitagliptin, or other compounds with high enantiomeric purity required in therapeutic applications [16,39].

Cellulases and laccases immobilized by adsorption are widely used in the textile industry [21]. Recently, laccase from *Trametes versicolor* was adsorbed on TiO_2_−ZrO_2_−SiO_2_ with nearly quantitative immobilization yields, and it was successfully used in the degradation of dyes from textiles [40]. This immobilization technique has been also used to adsorb the laccase onto polyvinylidene fluoride membranes to degrade pharmaceutical pollutants in wastewater treatment. This method allows for easy enzyme recovery and reuse, although desorption remains a challenge. Similarly, lipase has been immobilized on polypropylene membranes for biodiesel production, where it catalyzes the transesterification of oils [26].

Immobilization through simple adsorption or ionic binding (as described in the following paragraph) has the drawback of potential enzyme leaching in aqueous media, depending on the pH and ionic strength. This limitation restricts its use to water-free systems. To address this issue, a hybrid tailor-made immobilization method has been developed for membrane enzymes. Specifically, equine kidney γ-glutamyl-transpeptidase (ekGGT), a membrane enzyme used for the synthesis of γ-glutamyl amino acids, has been immobilized on a heterofunctional carrier with a high immobilization yield and activity recovery (93% and 88%, respectively). This carrier combines hydrophobic alkyl chains and aldehyde groups, enabling concurrent adsorption interactions that mimic the lipid environment of cellular membranes and covalent immobilization to stabilize the enzyme, thereby preventing potential leaching [41].

#### 2.1.4. Ionic Binding

Enzymes can be immobilized through ionic binding using an ion-exchange resin. The selection of a cationic or anionic resin is dictated by the enzyme’s net surface charge, which depends on its isoelectric point and the pH of the immobilization solution. The first large-scale industrial application of an immobilized enzyme was reported in 1969 by Tanabe Seiyaku, involving the use of an aminoacylase from *Aspergillus oryzae* immobilized by adsorption on DEAE-Sephadex (cross-linked dextran functionalized with diethylaminoethyl groups) for the synthesis of an l-amino acid [42].

As previously described, ionic immobilization presents similar drawbacks to adsorption immobilization. In this case as well, hybrid immobilization methods, combining ionic immobilization followed by covalent immobilization, have been developed for multimeric enzymes. For example, nucleoside phosphorylases from *B. subtilis* were immobilized onto Sepabeads coated with poly (ethyleneimine) (Sep-PEI) by ionic binding and, finally, the derivative was treated with polyaldehyde macromolecules (20% oxidized dextran) that, reacting both with the free amino groups of the enzyme and PEI, afforded a covalent multipoint cross-linking between the protein subunits and the support (76% immobilization yield) [43].

### 2.2. Classical Covalent Immobilization

Covalent immobilization requires the presence of two mutually reactive chemical groups on the enzyme and on a carrier surface (carrier-bond) or another protein molecule (carrier-free). Classical covalent immobilization strategies exploit the reactivity of endogenous functional groups present in the side chains of the amino acids of the enzyme. Table 1 shows some of the reactive groups present in naturally occurring amino acids that are used for covalent immobilization. Amines and thiols are both good nucleophiles and are the most widely exploited residues for covalent immobilization. Carboxylic acid groups have to be activated to make them reactive toward nucleophiles [13].

Among the classical methods of covalent enzyme immobilization, there are also strategies that do not require any support (CLEs, CLEAs, and CLECs, Figure 1). This topic has been extensively described and reviewed by Roger Sheldon [16,44] and will not be addressed in this review. It is worth highlighting that cross-linking has been adopted as a recent application for EIM. One key example is the immobilization of β-galactosidase on polyvinylidene fluoride membranes using glutaraldehyde (GA) cross-linking. This enzyme derivative is used in the production of galacto-oligosaccharides (GOS), which serve as prebiotic ingredients in functional foods. The cross-linking process significantly improves enzyme retention, although excessive reticulation may slightly reduce enzymatic efficiency due to steric hindrance. A similar strategy has been employed for lipase immobilized on alumina hollow fiber membranes, facilitating hydrolysis reactions in industrial settings. The high surface area and strong cross-linking bonds ensure prolonged activity, making this an attractive option for large-scale enzymatic processes [26].

#### Carrier-Bound

Unlike immobilization via cross-linking, covalent bonding involves attaching enzymes to support materials, such as porous silica, polyacrylamide, agarose, or porous glass, forming a robust and stable connection, minimizing leakage issues. The attachment of the enzyme to the support can occur by establishing multi-point or single-point covalent bonds between the functional groups on the enzyme’s surface and suitable reactive groups on the solid support (Table 2). Covalent immobilization mediated by the carrier involves modifying the polymer structure through support activation achieved by the addition of reactive molecules [18,45].

**Scheme 1:**
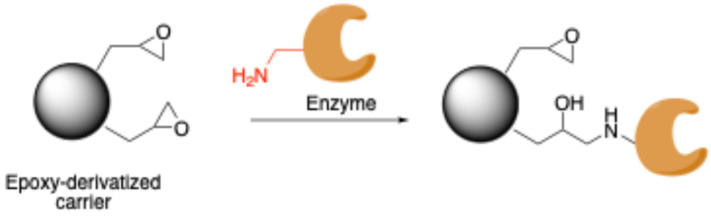
Covalent immobilization of enzymes to epoxy methacrylate resins.

Covalent immobilization by epoxy carriers involves the reaction of free amino groups, such as lysine residues, on the surface of enzymes. A straightforward method to achieve this is by exploiting the epoxy groups of functionalized resins (e.g., methacrylate carriers), which undergo nucleophilic attack at mild pH values (pH 8) by the available amino groups on the enzyme’s surface. This reaction leads to the formation of stable secondary amine bonds, effectively immobilizing the enzyme (Scheme 1). Oxirane groups are also able to react with various nucleophilic groups, in addition to primary amino groups, such as thiol groups, forming strong thioether bonds. However, the reactivity of epoxy groups is low and, generally, a long incubation time is required for immobilization (at least 24 h). First, the enzyme has to come in close contact with the carrier (adsorption) and, subsequently, the covalent bond is formed.

**Scheme 2:**
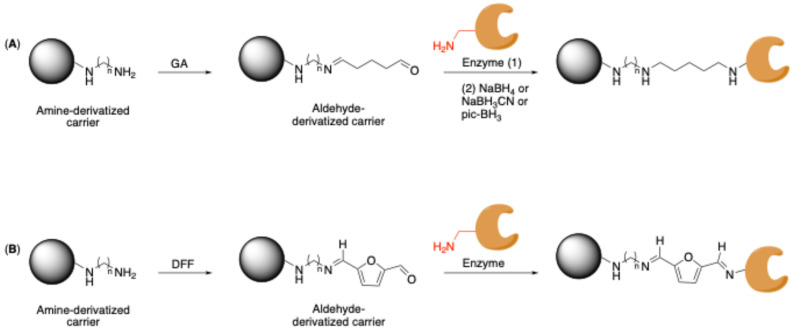
Immobilization of enzymes on amino functionalized carrier. (**A**) Derivatization with GA; (**B**) derivatization with DFF.

The amino groups on the functionalized carrier can react with the enzyme after pre-activation with glutaraldehyde (GA) [46] or other safer bifunctional reagents (e.g., 2,5-diformylfuran [47]) (Scheme 2). This process involves the interaction of the resulting aldehyde groups with the enzyme’s amino groups, generally at basic pH values (pH 10), leading to the rapid formation of Schiff bases, which are unstable under acidic conditions. These imines can subsequently be reduced (e.g., usually by NaBH_4_, NaBH_3_CN, or 2-picoline borane complex), resulting in irreversible enzyme immobilization, although this may carry the risk of diminishing the activity of the biocatalyst [16,18,45,48]. If 2,5-diformylfuran (DFF) is used, the imine bond is also stable at acidic pH values thanks to the conjugation of the formed imine bond with the furan ring, thus avoiding the reduction step [47].

Many examples of biotransformations catalyzed by enzymes immobilized on epoxy and amino carriers have been reported for the synthesis of compounds relevant to pharmaceutical and food fields [21].

**Scheme 3:**
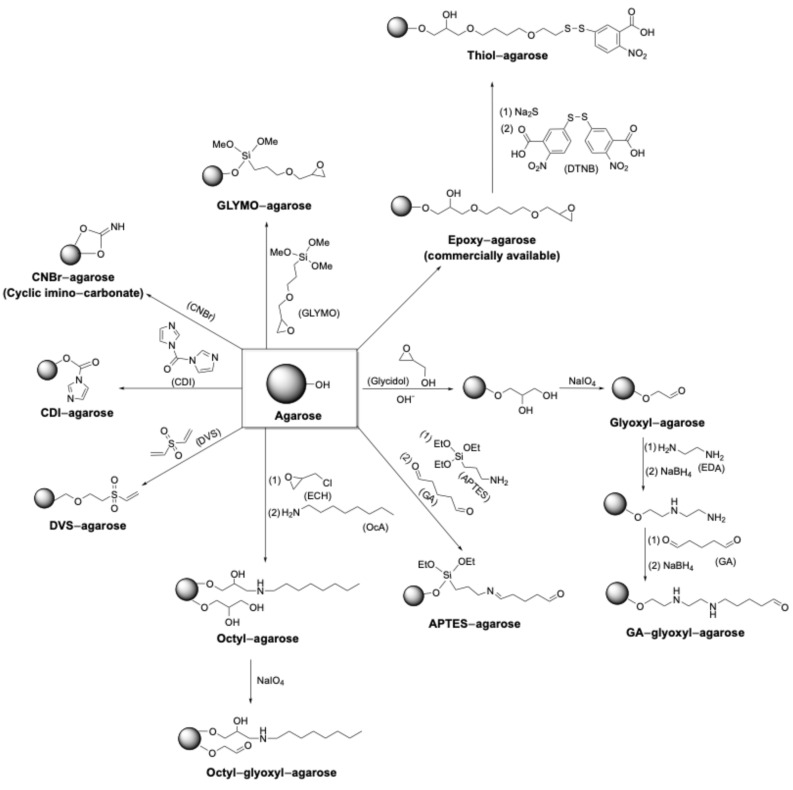
Chemistry of functionalized agarose. Legend: APTES = (3-aminopropyl)triethoxysilane; CDI = *N*,*N*′-carbonyldiimidazole; GLYMO = (3-glycidyloxypropyl)trimethoxysilane; CNBr = cyanogen bromide; DTNB = 2,2′-dinitro-5,5′-thiobenzoic acid; DVS = divinyl sulfone; ECH = epichlorhydrin; GA = glutaraldehyde; OcA = octylamine.

The hydroxyl groups present in polysaccharide-based carriers are very versatile and can be derivatized by a plethora of activations, allowing the covalent attachment of enzymes by different binding chemistries. Agarose is one of the most inert polymers that is widely used for enzyme immobilization after activation of hydroxyl groups (Scheme 3) [20,49,50,51,52,53,54].

Glyoxyl-agarose, glutaraldehyde glyoxyl-agarose, and APTES-agarose, with different spacer groups, react with the enzymes using the same binding chemistry described in Scheme 2. In fact, these derivatized carriers contain free aldehyde groups, enabling the multipoint covalent attachment of enzymes through Schiff base formation with the free amino groups of lysine residues. By varying the length of the spacer groups, the properties of the final enzyme derivative can be modulated since longer spacer arms allow an immobilized biocatalyst to be obtained with more flexibility as well as more distance from the carrier surface. Depending on the enzyme and the application, these properties can negatively or positively influence the outcome of the final derivative. Aldehyde-agarose-based carriers are likely the most commonly used supports for protein immobilization. Moreover, the enzymes immobilized on glyoxyl-agarose have been successfully applied to biotransformations under flow conditions [55,56,57,58].

As previously reported, the octyl-glyoxyl-agarose carrier has been successfully employed in the hybrid, tailor-made immobilization of membrane proteins [41].

The immobilization using GLYMO-agarose also occurs, as described in Scheme 1.

**Scheme 4:**
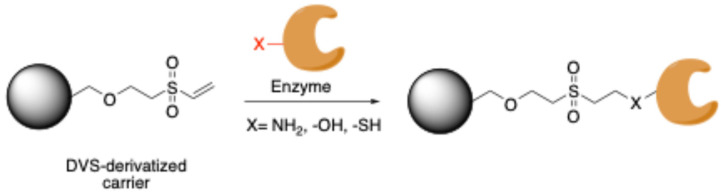
Immobilization of enzymes on DVS functionalized carrier.

Hydroxyl group activation with divinyl sulfone (DVS) can react mainly with imidazole and thiol groups of the amino acid side chain, but some reactivity with amino and phenol groups has also been observed depending on the pH [59]. DVS groups are much more reactive compared to epoxy groups, since they are able to covalently immobilize enzymes without requiring previous adsorption of the enzyme to the surface. Moreover, DVS supports can be used in a wide range of pH values compared to glyoxyl-agarose, which is generally employed at alkaline pH values. Moreover, compared to an aldehyde-based carrier, DVS-activation allows a stable enzyme–carrier linkage to be directly obtained without the need for the reduction step (Scheme 4). Different enzymes, such as ketoreductase P1-A04 from Codexis [60], as well as lysine cyclodeaminase from *Streptomyces pristinaespiralis* [61], were immobilized at mild pH on DVS-agarose through interaction with His residues obtaining good immobilization yields but unfortunately low recovered activity. This was attributed to a strong rigidification of the protein structures after covalent immobilization.

**Scheme 5:**
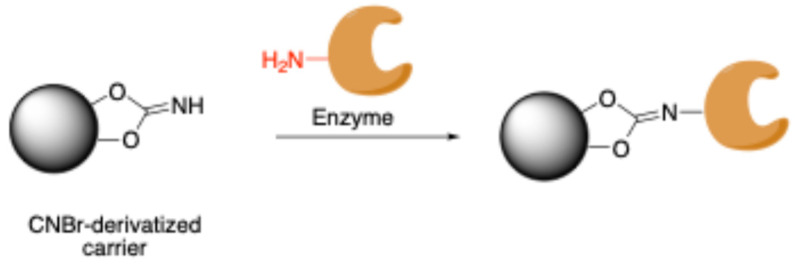
Covalent immobilization of enzymes to CNBr-agarose.

The hydroxyl groups of the agarose (vicinal diols) can react with cyanogen bromide (CNBr) to give the reactive cyclic imido-carbonate [62]. This primarily reacts with the *N*-terminal amino acid under mildly basic conditions, allowing for single-point immobilization (Scheme 5). CNBr-activated agarose has proven to be an effective carrier for the immobilization of native human epidermal growth factor (hEGF), ensuring proper orientation of the protein to prepare an active and flexible supported EGF for tissue engineering. The bioconjugate was obtained in 65% yield after 3 h [63].

**Scheme 6:**
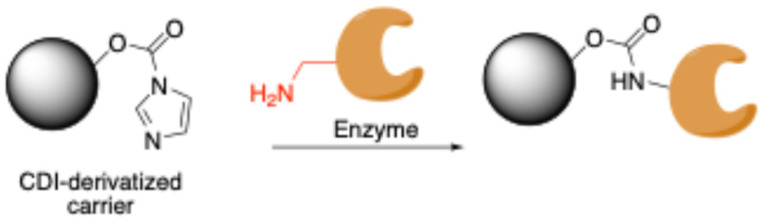
Covalent immobilization of enzymes to CDI-agarose.

Different immobilization binding chemistry can be used when *N*,*N*′-carbonyldiimidazole (CDI) is employed to activate the agarose. CDI is a highly reactive carboxylating agent that contains two acylimidazole leaving groups, which form reactive carbonyl groups on the hydroxyl support. This conjugate reagent has been successfully used in peptide synthesis and also in the immobilization of enzyme and affinity ligands in a chromatography matrix. The activation process of the carrier has to be performed in anhydrous media due to the susceptibility to hydrolysis of CDI [50]. Immobilization on agarose-CDI is another technique that enables multipoint enzyme-support interaction through the formation of carbamate bonds between the surface lysins of the enzyme and the activated support (Scheme 6). Few examples of enzyme immobilization via CDI-carrier are currently reported in the literature [64]. A very interesting example involves the conjugation of ovalbumin, used as a model antigen, onto magnetite nanoparticles for application as particulate vaccines [50].

The same binding chemistry described for immobilizing enzymes on different functionalized carriers is used for enzyme immobilization on membranes. In this case, chemical bonds can be present either on the surface or within the pores of the membrane. In most membranes, there are few naturally occurring functional groups that can be directly used for covalent bonding. For this reason, membrane pretreatment with functionalizing reagents is commonly applied for covalent immobilization using methods such as wet chemistry, UV activation, gamma irradiation, and plasma treatment. A notable example is β-galactosidase covalently attached to polyethersulfone membranes, functionalized with GA, for the continuous hydrolysis of lactose in the dairy industry. This system enables the production of lactose-free milk with an extended enzyme lifespan. However, the rigidity of covalent bonds may induce conformational changes in the enzyme, potentially reducing its catalytic efficiency. Another example is laccase immobilized on TiO_2_-coated membranes activated with APTES and then GA (activity recovery of 79%), used in pharmaceutical wastewater treatment. The enzyme is chemically linked to the modified membrane surface, allowing for long-term degradation of antibiotics such as erythromycin and tetracyclines. This immobilization strategy ensures minimal enzyme loss, making it highly effective in continuous processes [26].

## 3. Site-Specific Immobilization

**Figure 2:**
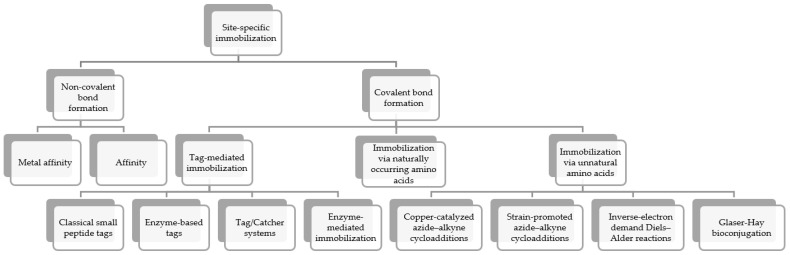
Site-specific immobilization techniques classification.

For some applications, an oriented immobilization protocol is highly desirable. In biocatalysis, optimal accessibility of the substrate to the active site of immobilized enzymes is highly sought. Being able to immobilize the biocatalyst with its active site oriented toward the solution through an oriented immobilization protocol allows higher catalytic efficiency and a homogeneous activity to be obtained compared to an immobilized biocatalyst by a conventional method, leading to a randomly oriented immobilization. In contrast, redox enzymes generally require their active site to be in close contact with the support surface to optimally receive or transfer electrons from or to the support [65]. The ability to form an oriented binding relies on unique chemical functionalities present on the surface of the enzyme that has to be immobilized. These unique chemical functionalities can be naturally present or synthetically introduced by engineering approaches into the amino acidic sequence of the enzyme. Few examples of naturally unique reactive groups occurring are reported in the literature. For example, glycosylated enzymes are characterized by the presence on their surface of covalently bound oligosaccharide units. These carbohydrate residues, if properly exposed on the surface of the enzyme and if not essential for the catalytic activity, can be used for a reversible covalent interaction with boronic acid derivatized carriers [66]. Different classes of enzymes have been immobilized via boronic acid-*cis*-diol interaction: horseradish peroxidase [67], lipase from *Candida antarctica* B [68], laccase from *Pleurotus ostreatus* [69]. Other examples rely on the presence of unique amino acid residues in the enzyme sequence. Pepsin contains one phosphoserine residue in its whole sequence (304 amino acids). This unique residue was selectively exploited for its immobilization on alumina [70]. Bromelain contains just one His residue (His158) in its whole amino acidic sequence (23.8 kDa). This unique residue was successfully exploited for its immobilization on Cu^2+^ derivatized iminodiacetic acid Sepharose 6B carrier [71]. However, since the presence of naturally occurring unique residues is quite rare, nowadays, the sequence of the enzymes can be easily engineered by the addition of tags at the *N*- or *C*-terminus or by the introduction of unique chemical functionalities into the sequence of the protein to allow a site-specific immobilization protocol. The possible engineering techniques can include the fusion of the *N*- or *C*-terminus of the protein sequence to genetically encoded functional tags [72], the use of post-translational enzyme-catalyzed protein modifications [73], the incorporation of naturally occurring amino acids by site-specific mutagenesis into a small area of the protein surface [65], the incorporation of unique unnatural amino acids into protein sequences by genetic methods such as amber codon suppression mutagenesis [74]. In this section, we will discuss in more detail the use of engineering techniques reported in the literature in order to achieve an oriented site-specific immobilization protocol for enzymes applied in biocatalysis. The site-specific immobilization approach can be divided into non-covalent and covalent strategies (Figure 2).

### 3.1. Site-Specific Non-Covalent Immobilization

Enzymes can be non-covalently bound in an oriented way onto a carrier via affinity interaction. The enzyme sequence must be engineered using recombinant techniques in order to introduce an affinity tag at the *N*- or *C*-terminus of the protein sequence that is able to recognize and bind specifically to its counterpart present on the immobilization carrier. Affinity immobilization techniques exploit the selectivity of specific interactions such as between polyhistidine and metal ions, (strept)avidin and biotin, antibodies and antigens, lectins and glycosylated macromolecules, nucleic acids and nucleic acid-binding proteins, hormones, and their receptors and many more [72]. However, these selective non-covalent immobilization strategies have mostly been borrowed from the protein purification area. Therefore, binding is usually reversible, making them sometimes inappropriate as an effective immobilization technique.

#### 3.1.1. Site-Specific Non-Covalent Immobilization via Metal Affinity

A versatile and widely used affinity tag is the polyhistidine tag (His-tag). The polyhistidine tag (6 to 10 histidine residues, typically) can be easily fused to the *N*- or *C*-terminus of the enzyme of interest thanks to the availability of several commercial expression vectors that include this tag, and the carrier has to be derivatized with chelating moieties (such as nitrilotriacetic acid or iminodiacetic acid) bearing divalent metal ions (Cu^2+^, Ni^2+^, Zn^2+^, Co^2+^) or trivalent metal ions (Fe^3+^). The choice of metal ions to be used for carrier derivatization is highly dependent on the application since the different metal ions have different affinity and specificity in binding His-tagged proteins.

**Scheme 7:**
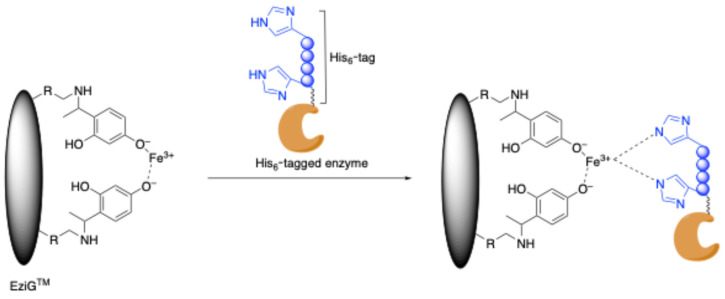
Immobilization of His_6_-tagged enzyme onto commercial EziG^TM^ support. The enzyme is immobilized onto the solid carrier through chelation of the metal ion by the imidazole moieties of the His residues.

This technique is widely used in the biocatalysis field to achieve a one-pot purification and immobilization protocol of the desired recombinant enzyme. Many enzymes have been immobilized via His-tag both for biocatalytic application and biosensor development on different carrier materials: cutinase [75], alcohol oxidase [76], ω-transaminases [77], ene-reductases [78], β-galactosidase [79], β-mannosidase [80]. The commercially available EziG^TM^ carriers by EnginZyme AB Sweden (Stockholm, Sweden) have been used for the immobilization of a broad range of enzymes for both batch and flow biocatalytic applications [76,81]. These carriers are based on glass beads (plastic-free) coated with an organic polymer and chelated Fe^3+^ ions, which enable the selective binding of His-tagged proteins directly from crude cell lysate allowing to achieve a one-pot purification and immobilization protocol (Scheme 7). EziG^TM^ is available in three versions with different surface hydrophobicity: Opal (hydrophilic), Coral (hydrophobic), and Amber (semi-hydrophilic) [76].

As already said, the fusion of histidine tags can only occur to the *N*- or *C*-terminus of recombinant proteins, limiting the possibilities of enzyme orientation. In a recent example, the surface of two dehydrogenases (i.e., ADH from *Bacillus stearothermophilus* and ADH from *Thermus thermophilus*) was engineered by adding His-clusters into regions not involved in the catalysis in order to drive the immobilization via coordination bond [82]. By varying the position and the histidine density of the clusters, a small library of enzyme variants was created and immobilized on different carriers functionalized with different densities of various metal chelates (Co^2+^, Cu^2+^, Ni^2+^, and Fe^3+^). The authors demonstrated that His-clusters can be as efficient as the conventional His-tags in immobilizing enzymes, recovering even more activity, and gaining stability against some denaturing agents. For some His-clustered variants, certain orientations lead to more active heterogeneous biocatalysts, whereas other orientations lead to more stable immobilized enzymes in comparison to their His-tagged counterparts immobilized on the same carriers.

**Scheme 8:**
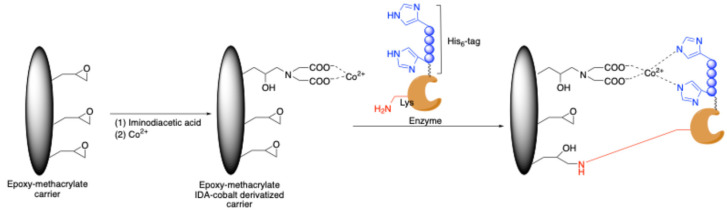
Immobilization of His_6_-tagged enzyme by mixed binding chemistry. Preparation of metal chelate epoxy carrier: commercially available methacrylic epoxy carrier is derivatized with iminodiacetic acid to introduce a few metal chelate sites. The IDA carrier is then incubated with cobalt salts to obtain metal chelate epoxy carriers. The enzyme comes first in close contact with the carrier through affinity interaction. Afterward, immobilization conditions are changed in order to allow the covalent bond formation between the remaining epoxy groups of the carrier and “classical” amino-bearing groups present on the enzyme surface (e.g., lysins).

However, the coordination interaction is reversible and can be disrupted by the addition of competitive ligands like imidazole or by metal chelators (e.g., ethylenediaminetetraacetic acid). The reversibility of the binding can be an advantage for certain applications where reusability of the carrier is required (e.g., purification of recombinant proteins from cell lysates or biochips production) but can be a disadvantage if stability and shelf life are an issue for the desired final immobilized derivatives. To enhance the stability of the binding, His-tag interaction can be combined with covalent binding through classical immobilization by designing a heterofunctional carrier (Scheme 8). In this case, a two-step immobilization process will occur: first, the protein will come in close contact with the carrier in an oriented way by affinity His-tag interaction followed by covalent bond formation with classical chemical groups present on the surface of the carrier (e.g., epoxy groups). [61,83]. More recently, a new covalent site-specific method was also developed for His-tagged proteins onto a vinyl sulfone derivatized surface (see later) [84].

#### 3.1.2. Site-Specific Non-Covalent Immobilization via Biotin-(Strept)Avidin Interaction

**Scheme 9:**
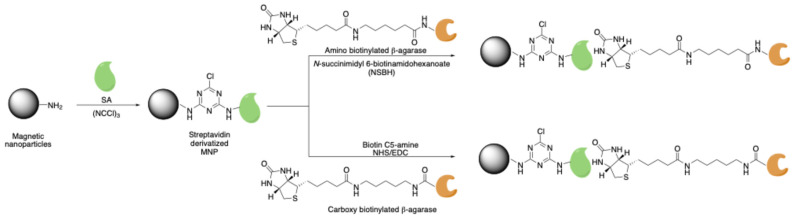
Immobilization of β-agarase through biotin-streptavidin interaction. Magnetic nanoparticles Fe_3_O_4_ (MNP) were prepared using the solvothermal reduction method and subsequently derivatized with cyanuric acid (NCCl)_3_ and streptavidin (SA). β-Agarase was chemically derivatized with biotin through its amino or carboxyl groups.

The biotin-(strept)avidin interaction is one of the most specific and stable non-covalent interactions with a dissociation constant about 10^3^ to 10^6^ times higher than an epitope–antibody interaction. Biotinylated enzymes can be immobilized onto (strept) avidin-coated surfaces. To perform this immobilization protocol, the enzyme to be immobilized has to be engineered in order to introduce on its surface the biotin moiety. At the same time the carrier has to be engineered and derivatized with (strept)avidin (53 kDa). Biotinylated enzymes can be prepared through chemical methods that generally conjugate biotin molecules to Lys residues of the protein. Pullulanese, an important biocatalyst in the food industry, was immobilized onto magnetic nanoparticles based on the specific recognition between biotin and streptavidin. Pullulanase was chemically biotinylated using biotin-*N*-hydroxysuccinimide, and the magnetic nanoparticles were prepared and functionalized with streptavidin using cyanuric chloride. The immobilized pullulanase retained high levels of activity (85%) and exhibited significantly improved pH and thermal stability as compared to the soluble enzyme. Moreover, the excellent recyclability of the immobilized biocatalyst was also reported, retaining more than 74% of its initial activity after eight consecutive reaction cycles [85]. In another example, β-agarase was chemically biotinylated through two different activation procedures: (i) the amino groups of β-agarase were reacted with *N*-succinimidyl 6-biotinamidohexanoate, (ii) the carboxyl groups of β-agarase were first reacted with 1-(3-dimethylaminopropyl)-3-ethylcarbodiimide hydrochloride and *N*-hydroxysuccinimide to obtain amine-reactive terminus and, then coupled with biotin C-5-amine (Scheme 9). Results showed that, compared with the soluble enzyme, the stability and substrate affinity of immobilized β-agarase preparations through amino or carboxyl activation were both significantly improved. However, the amino-activated immobilized β-agarase showed higher thermostability and catalytic efficiency than the carboxyl-activated immobilized β-agarase [86].

One limitation in applying a chemical biotinylation procedure is the difficulty in controlling both the extent and the site of biotinylation since most proteins have more than one residue on their surface that can be biotinylated. Thus, the overall orientation of the immobilized enzyme cannot be precisely controlled, but it depends on which biotin(s) interacts with the strept(avidin) probe. Nowadays, it is possible to apply a controlled biotinylation procedure by fusing the *N*- or *C*-terminus of the enzyme that has to be immobilized with a 75 amino acid biotin carboxyl carrier protein (BCCP) module from *E. coli* acetyl CoA carboxylase [87]. The BCCP tag is recognized and can be biotinylated in vivo or in vitro by *E. coli* biotin ligase/synthetase (BirA). The ligation reaction catalyzed by BirA can also be applied for the biotinylation of other smaller tags such as the Avitag, a tag of 15 amino acids that is rapidly biotinylated by BirA [88]. β-Galactosidase from *E. coli* was fused at its *N*-terminus with a polypeptide tag that was site-specifically biotinylated by biotin ligase during the post-translational modification process in *E. coli*. Subsequently, the biotinylated enzyme was incubated in the presence of poly(ether sulfone) membranes derivatized with avidin. The activity of this site-specific immobilized enzyme was two-fold higher compared to that of the commercially available non-specific biotinylated enzyme that was immobilized in a random way on the same carrier [89]. The *C*-terminus of β-glucosidase from *B. licheniformis* was fused with a biotin acceptor peptide and co-expressed with BirA biotin ligase. The immobilized enzyme showed improved thermal stability compared to the soluble enzyme and good recyclability (after nine cycles of reaction, the biocatalyst could retain 89% of its initial activity) [90].

### 3.2. Site-Specific Covalent Immobilization

The site-specific covalent immobilization procedures rely on the introduction of a unique bio-orthogonal chemical group or a sequence tag in the enzyme at a site-specific location that is able to form a covalent bond with a mutually reactive group present on a carrier. The site of enzyme engineering has to be strategically chosen in order to not impact the conformation of the enzyme and, thus, its catalytic activity. Three different strategies can be applied to achieve site-specific covalent immobilization as depicted in Figure 2: (i) tag-mediated immobilization, (ii) immobilization via naturally occurring amino acids positioned in specific regions of the protein, and (iii) immobilization via unnatural amino acids.

#### 3.2.1. Tag-Mediated Bio-Orthogonal Covalent Immobilization

Covalently binding tags allow a rapid, highly specific, and irreversible binding of recombinantly tagged proteins to specific ligands that can be covalently or non-covalently attached to the solid carriers. As depicted in Figure 2, covalent immobilization tag systems can be based on (i) classical small peptide tags (His-tag, Cys-tag); (ii) self-labeling protein tags constituted by enzymes that are able to catalyze their covalent attachment to specific synthetic ligands (HaloTag (Promega), SNAP-tag (New England Biolabs), CLIP-tag (New England Biolabs)); (iii) bacterial domains containing intramolecular isopeptide bonds split into two parts, a Tag peptide, and a Catcher protein; and (iv) small peptides that are recognized in vitro by enzymes able to mediated a bond formation (e.g., sortase, transglutaminase). The engineering of enzymes by fusing a specific tag sequence can occur just to the *N*- or *C*-terminus of recombinant proteins, limiting the possibilities of enzyme orientation. However, generally, these tags are small molecules that do not affect the expression and solubility of the engineered enzymes, and sometimes, these tags can also enhance the solubility of recombinant proteins. Moreover, the fusion of tags does not require the availability of the three-dimensional structure of the protein.

##### Classical Small Peptide Tags

**Scheme 10:**
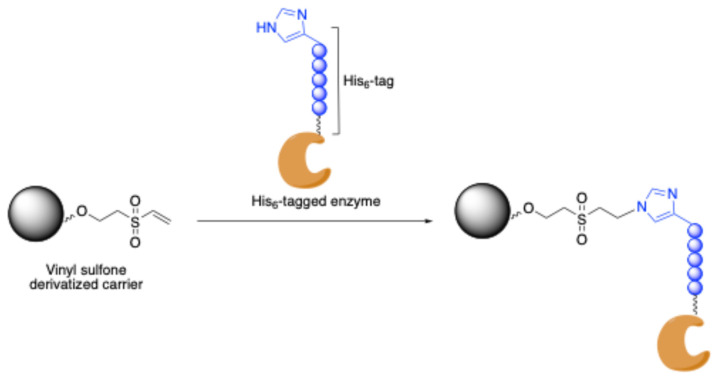
Covalent site-specific immobilization of His-tagged proteins onto vinyl sulfone-bearing surface. The imidazole group of the last His residue of His_6_-tag is deprotonated at neutral pH and can undergo Michael-type addition by vinyl sulfone groups present onto the carrier.

As already anticipated, a new covalent site-specific method was developed for the immobilization of proteins through His-tag [84]. Two model His-tagged proteins (HaloTag-6xHis and anti-HER2 Fab-6xHis) were conjugated onto surfaces derivatized with vinyl sulfone groups through a covalent bond (Scheme 10). Other than covalent binding, the newly developed protocol allowed us to obtain high protein loading and high binding specificity.

**Scheme 11:**
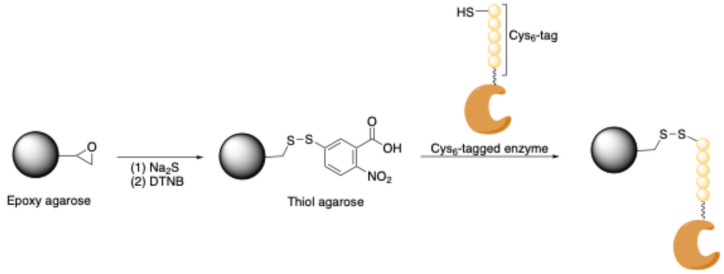
Covalent site-specific immobilization of Cys-tagged proteins onto thiol agarose. The commercially available epoxy agarose was derivatized with the thiol group in two steps: overnight incubation with 50 mM Na_2_S in 100 mM NaHCO_3_ pH 10 and subsequently incubation of 3 h with 10 mM 2,2′-dinitro-5,5′-thiobenzoic acid (DTNB). The Cys-tagged enzyme was covalently bound to the carrier by thiol exchange.

Enzymes with a tag consisting of cysteine repeats (Cys-tag) genetically fused at one terminus can be covalently attached to a solid support by disulfide bond formation. Purine nucleoside phosphorylase from *Halomonas elongata* (HePNP) with a Cys_6_-tag was immobilized on agarose beads activated with thiol (SH) groups (Scheme 11) and used for the synthesis of nucleosides analogs in the flow system [91]. The resulting immobilized HePNP on SH-agarose was shown to be as active as the enzyme covalently immobilized on epoxy/Co^2+^-activated agarose and more active than HePNP immobilized on other carriers (silica or methacrylate) with different binding chemistries (epoxy/Co^2+^, aldehyde). This immobilization strategy allowed an oriented and selective immobilization protocol to be obtained compared to the other tested immobilization strategies. Moreover, the covalent but reversible binding chemistry allowed the reusability of expensive agarose beads in a second cycle of enzyme immobilization after inactive enzyme removal with DTT treatment (50 mM).

##### Self-Labeling Protein Tags

**Scheme 12:**
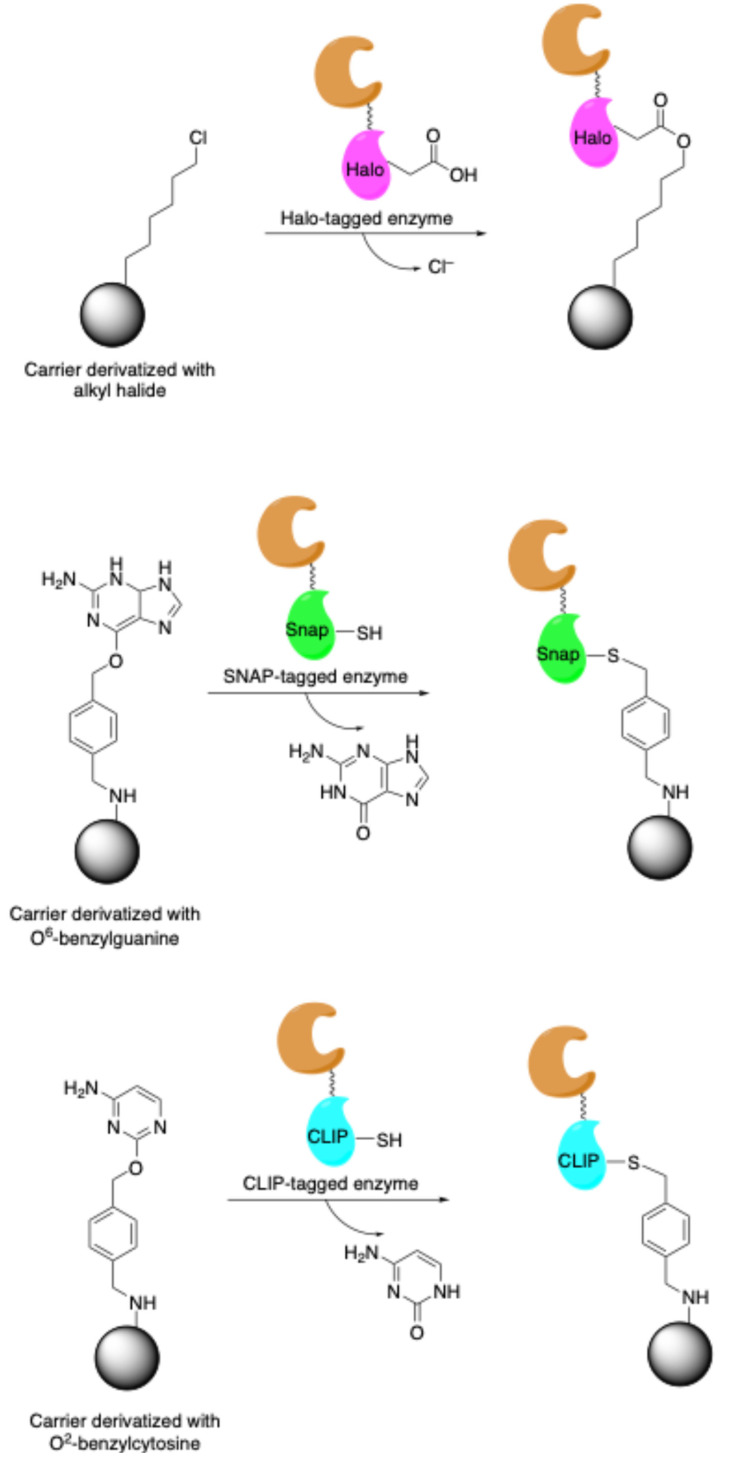
Site-specific covalent enzyme immobilization by fusion to self-labeling tags: HaloTag (33 kDa) forms a covalent bond through nucleophilic displacement of halides from alkyl halide derivatized carriers, SNAP-tag (20 kDa) reacts with O^6^-benzylguanine derivatized carriers while CLIP-tag (also 20 kDa) reacts with O^2^-benzylcytosine derivatized carriers.

Self-labeling protein tags are small proteins, typically less than 40 kDa, able to catalyze the self-covalent attachment to a small-molecule probe that is functionalized with a bio-orthogonal linker [92]. Self-labeling protein tags include the HaloTag (33 kDa), SNAP-tag (22 kDa), and CLIP-tag, an engineered variant of the SNAP-tag (Scheme 12).

Among the tags based on enzymes that are able to catalyze their covalent attachment to specific synthetic ligands, the HaloTag^TM^ is the most used for enzyme immobilization [72]. This system is based on a modified haloalkane dehalogenase from *Rhodococcus rhodocrous* that specifically recognizes terminal chloroalkane ligands, with which it establishes a stable covalent ester bond via an aspartate residue present in the active site (Scheme 12). The thiamine diphosphate-dependent benzaldehyde lyase from *Pseudomonas fluorescence* (PfBAL) fused with HaloTag was rapidly purified and immobilized from crude extracts in high purity. The immobilized biocatalyst was stable at 4 °C for months and was successfully reused in several repetitive batches for the carboligation of aggressive aldehydes (benzaldehyde and acetaldehyde) toward (*R*)-hydroxy-1-phenylpropan-1-one [93]. Using the same strategy, benzoylformate decarboxylase from *Pseudomonas putida* (PpBFD) was combined with alcohol dehydrogenase from *Lactobacillus brevis* (LbADH) in a continuous enzymatic cascade. Both enzymes were selectively purified and immobilized on Halolink Resin^TM^ from crude cell extracts directly in plug-flow reactors. The system resulted in the formation of the target product (1*S*,2*S*)-1-phenylpropane-1,2-diol with high conversion rates and stereoselectivity (up to 99% and 96%, respectively), and high space-time yields (up to 1850 g L^−1^ d^−1^) [94]. Recently, three Fe(II)/α-ketoglutarate-dependent dioxygenases (CaKDO, CpKDO, FjKDO) were easily purified from cell lysates and covalently immobilized by the HaloTag system [95]. The recombinant production and immobilization of KDO are quite challenging due to their low solubility during expression and instability once purified. The HaloTag-based immobilization allowed a rapid concentration of the enzymes from cell-free extracts on the carrier surface with high residual activity and improved stability. The increase in the stability enabled the desired biotransformation to be performed using high substrate loading without the generation of any side products and the recycling of the biocatalysts. Moreover, the immobilized biocatalysts were also used in preparative lab-scale biotransformations, achieving product titers of 16 g L^−1^ (3*S*)-hydroxy-l-lysine (CaKDO) and (4*R*)-hydroxy-l-lysine (FjKDO), respectively, starting from 100 mM l-lysine. Additionally, a Halo-tagged immobilized lysine decarboxylase from *Selenomonas ruminantium* was utilized to convert the (3*S*)-hydroxy-l-lysine produced by CaKDO into (2*S*)-hydroxy-cadaverine in a 15 mL consecutive batch reaction and without intermediate product purification.

##### Catcher/Tag Systems

**Scheme 13:**
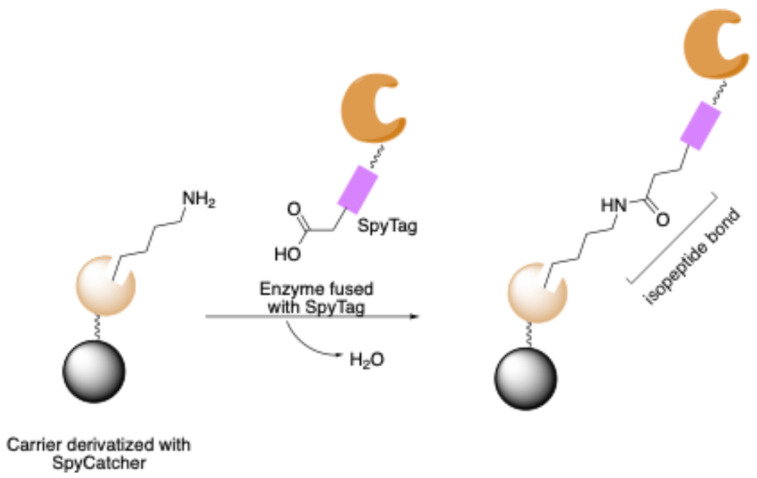
Site-specific covalent enzyme immobilization by SpyTag/SpyCatcher. The formation of the isopeptide bond between a lysine in SpyCatcher (Lys31) and an aspartic acid in SpyTag (Asp117) is spontaneous.

Another emerging immobilization approach in biocatalysis is the SpyTag/SpyCatcher system. The SpyTag/SpyCatcher pair is derived from the splitting and engineering of the CnaB2 domain of the fibronectin-binding protein (FbaB) from *Streptococcus pyogenes*. The SpyTag is constituted by the *C*-terminal beta strand of CnaB2 (13 aa) containing a reactive aspartic acid (Asp117), while the SpyCatcher (116 aa) is constituted by the rest of the beta strands containing a reactive lysine (Lys31). When the tag, generally fused to the recombinant enzyme or sometimes present onto the carrier, comes in close contact with its corresponding catcher, present on the carrier surface or fused to the recombinant enzyme, the interrupted intramolecular isopeptide bond is spontaneously reconstituted by forming a stable covalent bond (Scheme 13) [96]. The original SpyTag/SpyCatcher pair was further improved in order to achieve faster reaction rates (SpyTag002/3:SpyCatcher002/3) when compared to the original pair [97]. Furthermore, other Tag/Catcher pairs were developed by finding orthogonal proteins from different bacterial species, such as the SnoopTag/SnoopCatcher [98] and the SdyTag/SdyCatcher [99]. Additionally, an engineered non-reactive SpyCatcher variant (SpyDock) was developed in order to allow an on-demand reversible affinity binding [100]. For now, this system, called Spy&Go, has been employed for the purification of SpyTagged proteins, but it could also be exploited for enzyme immobilization.

The industrial enzyme glutaryl-7-aminocephalosporanic acid acylase (GA) was fused with SpyTag. After overexpression and purification, the fusion enzyme SpyTag-GA was loaded onto a SpyCatcher-derivatized carrier. A high immobilization efficiency (91%) and activity recovery (86%) were achieved in a very short time compared to the classical epoxy-mediated immobilization (immobilization efficiency = 29% and activity recovery = 3%) after 2 h of incubation. Using the same technology, the immobilization of SpyTag-GA was attempted directly from *E. coli* cell lysates. Also in this case, the SpyTag-GA was immobilized on the SpyCatcher-derivatized carrier in high yields (86%) and with high activity recovery (91%) compared to the epoxy-mediated immobilization samples, for which the immobilization efficiency and activity recovery of SpyTag-GA were only 25% and 3%, respectively [101]. In another example, d-allulose 3-epimerase (DAEase) from *Clostridium cellulolyticum* was successfully immobilized via covalent attachment on SpyCatcher-derivatized carrier through Spy chemistry and employed for the synthesis of d-allulose from d-fructose. The immobilized derivative maintained 80% of activity after seven consecutive cycles and 25 days of storage [102]. Finally, peptide-*N*-glycosidase F (PNGase F) fused to the SpyCatcher domain was site-specifically and covalently immobilized onto magnetic nanoparticles derivatized with SpyTag. The final biocatalyst exhibited good thermal stability and deglycosylation activity of different glycoproteins releasing various types of glycans (high-mannose, sialylated, and hybrid). Furthermore, after five reaction cycles, the immobilized enzyme retained 78% of the initial activity [103].

##### Enzyme-Mediated Immobilization Driven by Tag Sequences

**Scheme 14:**
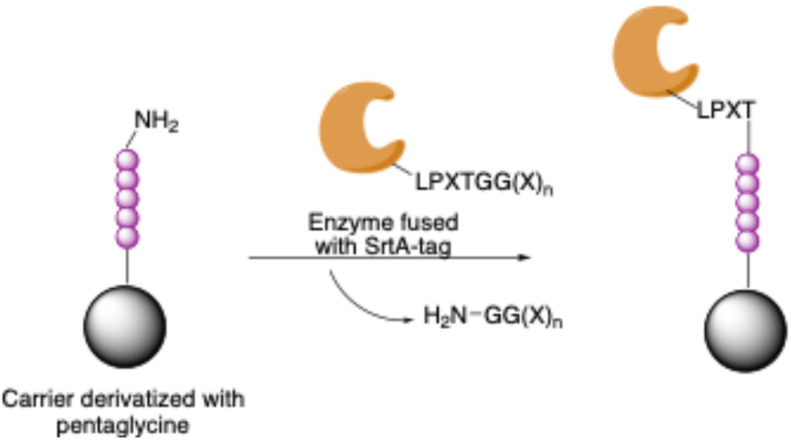
Site-specific covalent enzyme immobilization mediated by Sortase A. Instead of pentaglycine, the solid carrier can be derivatized with substrate nucleophiles that consist of a smaller number of glycine residues or simple amine groups. Alternatively, the LPXTG recognition sequence can be conjugated to the solid carrier, and the protein of interest can be tagged with an *N*-terminal oligoglycine motif.

In addition to the self-ligating systems, the covalent bond formation between the enzyme and the carrier can be mediated by an enzyme in vitro (enzyme-mediated immobilization). The transpeptidase Sortase A (SrtA) generally recognizes the LPXTG motif (where X is any amino acid except cysteine). SrtA cleaves the amide bond between the threonine and glycine in the LPXTG motif by forming a thioester intermediate, which can then be displaced by an amine nucleophile, typically the free amino group of a polyglycine substrate, forming a stable peptide bond. SrtA can be used as a sort of molecular “stapler” that mediates site-specific cross-linking between proteins fused with the LPXTG motif at their *C*-terminus and an amine group (or glycinamide motif) present on the carrier (Scheme 14) [104]. SrtA can be simply produced in-house in *E. coli* in large amounts, making this immobilization technique cost-effective.

*Thermobifida fusca* YX β-glucosidase (BGL) and *Streptococcus bovis* 148 α-amylase (AmyA) were successfully immobilized via SrtA-mediated transpeptidation on polystyrene nanoparticles derivatized with a tri-glycine tag. Both immobilized BGL and AmyA exhibited a 3.0- or 1.5-fold, respectively, higher activity compared to the same enzymes immobilized by classical (random) covalent immobilization. Moreover, the two biocatalysts were able to retain more than 80% of initial activity after 10 consecutive reaction cycles, suggesting good recyclability [105]. In another study, the use of graphene-oxide carriers functionalized simply with ethylenediamine as an amine donor group in sortase-mediated immobilization was demonstrated to be more effective than pentaglycine-derivatized graphene-oxide carriers. Silica-based carriers derivatized with pentaglycine were more effective than amine-functionalized silica [106]. These results suggested that the type of amine nucleophile used for the derivatization of the carrier and the type of carrier have to be carefully evaluated since they can affect the SrtA-mediated immobilization process, thus altering the final properties of the immobilized derivative. Based on these results, *Candida antarctica* lipase B (CalB) was covalently immobilized on the surface of amine-functionalized graphene oxide (GO) nanoparticles by SrtA-mediated immobilization [107]. Almost 60% of CalB was immobilized on the amine-derivatized support from crude extract after 16 h of incubation with an activity recovery of 132%, probably due to the uniform orientation of the enzyme molecules on the carrier that facilitates the accessibility of substrates to the active sites. Moreover, the oriented immobilized derivative was employed in the hydrolysis of fish oil, showing a 2.5-fold higher selectivity in the release of cis-5, 8, 11, 14, and 17-eicosapentaenoic acid compared to the immobilized derivative obtained by random immobilization. By using this strategy, two unstable membrane-bound glycosyltransferases were immobilized on amino-derivatized carriers [108]. Immobilized recombinant human β1,4-galactosyltranseferase or recombinant *Helicobacter pylori* R1,3-fucosyltransferase were used successfully in the repetitive synthesis of Lewis X antigen.

Microbial transglutaminases (mTG) can be used alternatively to SrtA to mediate an oriented and covalent immobilization of glutamine-tagged enzymes onto amine-derivatized solid carriers [109]. Over the years, different highly reactive Q-tags for the *Bacillus subtilis* mTG [110] and new mTG with high substrate specificity [111] were discovered, providing new tools for mTG-mediated bioconjugation. The alkaline phosphatase (AP) from *E. coli* tagged with a hexapeptide containing one glutamine residue (MLAQGS) was covalently and site-specifically immobilized by transglutaminase mediated immobilization onto a polystyrene surface physically coated with β-casein or bovine serum albumin displaying reactive lysine residues [112] as well as onto aminated magnetic particles [113]. Immobilization efficiency was affected by the type of amine group present on the surface magnetic particles. The derivatization of the carrier with diethyleneglycol bis (3-aminopropyl)ether (DGBE) allows the non-specific physical adsorption of the enzyme on carrier surface to be reduced and, thus, only the site-specific covalent immobilized enzyme to be obtained. The immobilized AP exhibited 93% of the initial activity after 10 rounds of recycling. The biotin ligase BirA from *E. coli*, frequently used for in vitro biotinylation of recombinant proteins, was immobilized onto amine-modified magnetic microsphere by mTG-promoted condensation [114]. The site-specifically immobilized BirA exhibited approximately 95% enzymatic activity of the soluble BirA, and no significant loss in activity after 10 rounds of recycling was observed. In addition, the immobilized BirA could be easily recovered from the solution via a simple magnetic separation. Thus, the immobilized BirA represents a robust biocatalyst of general use for efficient and economically feasible in vitro biotinylations. An engineered enterokinase fused at the *C*-terminus with the MLAQGS tag was site-specifically immobilized onto amine-modified magnetic nanoparticles via mTG-catalyzed bioconjugation for the development of a reusable biocatalyst [115]. Upon the site-specific immobilization, approximately 90% enterokinase enzymatic activity was retained, and the biocatalyst exhibited more than 85% of initial enzymatic activity regardless of storage or reusable stability over a month. The developed biocatalyst was further applied to remove the His-tag from the triplet of glucagon-like peptide-1 (GLP-1), showing remarkable reusability without a significant decrease in enzymatic activity after 10 reaction cycles. PNGase F was fused at the *N*-terminus with a WALQRPH tag that allowed a soluble expression of the enzyme and its immobilization via mTG-catalysis onto amine-modified magnetic particles [116]. The immobilized derivative exhibited the same *N*-deglycosylation activity as its soluble counterpart, as well as excellent stability and good reusability (79% residual activity after five reaction cycles), thus making it an efficient tool for *N*-glycan analysis.

#### 3.2.2. Sequence Engineering-Mediated Bio-Orthogonal Covalent Immobilization

Another strategy that allows greater versatility in enzyme orientation is the engineering of the sequence of the target enzyme by the introduction of natural amino acids or unnatural amino acids in specific regions of the protein. In order to apply this strategy, the three-dimensional structure of the enzyme (or some analogous one) should be resolved in order to introduce these modifications in a rational way. Moreover, it has to be taken into consideration that these modifications can interfere with the expression/stability of the recombinant-engineered enzyme.

##### Incorporation of Naturally Reactive Occurring Amino Acids by Site-Specific Mutagenesis

Enzyme immobilization can be oriented by the incorporation of naturally reactive occurring amino acids by site-specific mutagenesis into the biocatalyst sequence. Naturally, amino acids generally exploited also for random immobilization protocols (e.g., Lys or Cys) can be introduced on the protein surface by site-directed mutagenesis both as extra residues localized just on a desired region of the protein surface or as unique residues.

The addition of extra Lys residues localized in a specific region of the protein surface can allow an oriented immobilization protocol to be achieved by forcing the enzyme to interact with the carrier, mostly with a small enriched Lys surface area. This strategy generally requires the replacement of several amino acids present on a small surface area of the protein, and at the same time, the deletion of other lysine residues localized in areas that are not of interest could be necessary. However, introducing several mutations in a small surface area can negatively affect protein stability. Thus, the effect of the substitution is not easy to predict. The catalytic activity of the immobilized Penicillin G acylase (PGA) from *E. coli* on aldehyde agarose was improved by designing different engineered protein variants containing extra lysine residues on different regions of the protein since 72% of the wild-type PGA was immobilized on the carrier mainly through the Lys residues positioned on the same side of the active site, thus blocking the entrance to the bulky β-lactams substrates. The variant 3xLys-PGA, possessing three extra Lys residues alternating with three Gly residues at the end of the β chain, on the opposite site of the active site, showed better accessibility of the substrates since 63% of its active site was facing the reaction medium [117]. Following the same strategy, the immobilization yield of xylanase from *Chaetomium globosum* onto SiO_2_ nanoparticles was improved up to 99% compared to wild-type enzyme by designing a penta-mutated variant (Asn172Lys-His173Lys-Ser176Lys-Lys133Ala-Lys148Ala). The wild-type enzyme showed low immobilization yield due to a lack of lysine residues present on its surface. By applying a mutation energy-based in silico screening approach and some rational biochemical evaluations of surface residues, three residues were selected for lysine substitution: Asn172, His173, and Ser176, while two naturally occurring lysins 133 and 148, which were located near the active site region were mutated to alanine [118].

A single cysteine residue was introduced, using site-directed mutagenesis, into the PGA at six different regions of the enzyme that were rich in Lys residues. The different variants were immobilized on a disulfide-containing carrier through covalent immobilization, exhibiting excellent stability and selectivity in the synthesis of cephalosporines derivatives through additional protein rigidification. The replacement of Gln380 with Cys produced an immobilized derivative that was 30-fold more stable than the soluble preparation toward heat and organic co-solvents and preserved 90% of the initial activity [119].

##### Incorporation of Unnatural Amino Acids

**Scheme 15:**
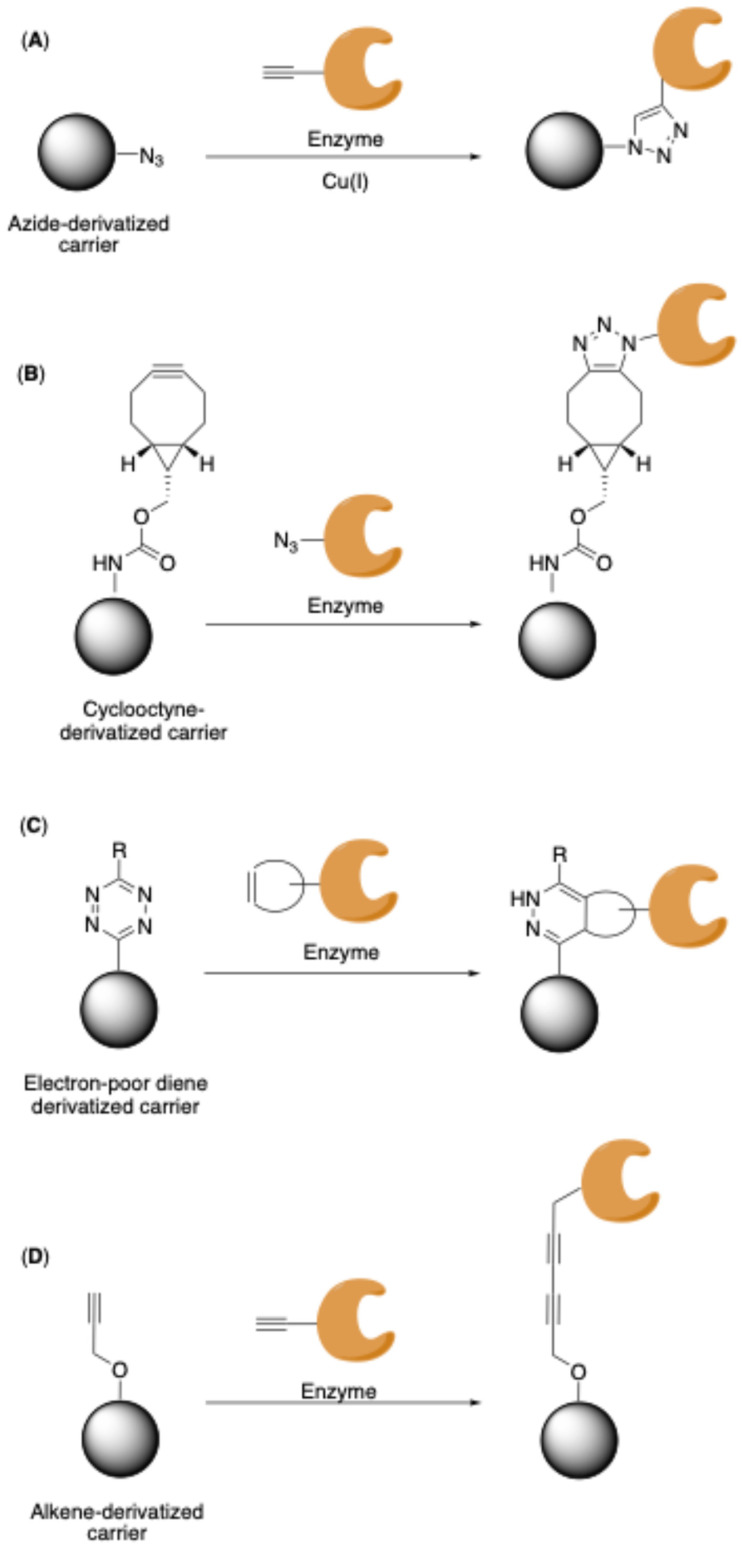
Site-specific covalent immobilization mediated by unnatural amino acids. (**A**) Cu(I)-catalyzed azide–alkyne cycloaddition (CuAAC) reaction, (**B**) strain-promoted azide–alkyne cycloaddition (SPACC) reaction, (**C**) inverse electron-demand Diels–Alder (IEDDA) reaction, (**D**) Glaser–Hay reaction.

The incorporation of unnatural amino acids with unique reactive groups into the enzyme sequence at a single site or multiple sites is an elegant strategy to achieve high precision in covalently bound formation between an enzyme and a carrier. Genetic code expansion can be used to insert a plethora of unnatural amino acids into the protein sequence. Based on amino acid type, a variety of covalent ligation reactions can be applied. Among all, copper-catalyzed azide–alkyne cycloadditions, strain-promoted azide–alkyne cycloadditions, and inverse-electron-demand Diels–Alder and Glasser-Hay reactions were applied for enzyme immobilization onto solid carriers (Scheme 15).

##### Copper-Catalyzed Azide–Alkyne Cycloadditions (CuAAC)

The copper-catalyzed Huisgen 1,3-dipolar cycloaddition of azides and alkynes is one of the best-known click reactions (Scheme 15A). Several Cu(I) sources can be used to catalyze the reaction, but preferably, the catalyst is prepared in situ by the reduction of Cu(II) salts [120]. This reaction is usually performed in aqueous buffer to produce a stable and inert 1,4-triazole. Different variants of T4 lysozyme containing *p*-propargyloxyphenylalanine (pPa) at different positions (Thr21, Lys35, Asn53, Leu91, Lys135, and Lys162) were produced by amber stop codon strategy and immobilized on superparamagnetic beads [121]. The activity of the immobilized enzyme was shown to vary depending on the site of conjugation: three variants in which the insertion of unnatural amino acid pPa were introduced far from the active site, Asn53pPa, Lys135pPa, and Lys162pPa, maintained high activity (70–85%) while the other two variants, Thr21pPa and Lys35pPa, showed a dramatic decrease in activity (<10% for Thr21pPa variant). The Leu91pPa variant had the highest retained activity of all the after immobilization and was further investigated. The site-specific immobilization of this variant enhanced its stability toward freeze-thaw cycles (80% retained activity) and the denaturant urea when compared to the enzyme immobilized in a random manner on epoxy-modified beads as well as soluble lysozyme.

The use of a copper catalyst increases the azide-alkyne click reaction rate, but the formation of reactive oxygen species generated during these reactions has been well documented to negatively affect the structure and functional activity of proteins.

##### Strain-Promoted Azide-Alkyne Cycloadditions (SPAAC)

Strain-promoted azide-alkyne click reaction is a metal-free [3+2] cycloaddition reaction widespread used for enzyme immobilization. Cyclooctyne is the smallest stable cyclic alkyne with a 17° distortion from the preferred linear alkyne geometry, thus allowing a sufficient ring strain able to promote a metal-free cycloaddition of azides (Scheme 15B). This reaction generally proceeds at a slow rate compared to the copper-catalyzed reaction and generally requires high protein concentrations and overnight incubations. These conditions can promote nonspecific protein adsorption, protein aggregation, and loss of enzyme activity.

Amber codon suppression was used to replace five tyrosine residues (50, 137, 243, 274, and 355) with 4-azido-l-phenylalanine in *Geobacillus* sp. SBS-4S lipase [122]. The variants were conjugated to mesocellular siliceous foams derivatized with cyclooctene groups using the SPAAC reaction. The variant AzPhe-Lip243 showed higher catalytic activity and higher thermostability compared to the wild-type enzyme conventionally immobilized by using glutaraldehyde. In another example, 4-azido-l-phenylalanine, was incorporated into five specific sites of a laccase from *Streptomyces coelicolor* (SLAC) and immobilized onto multi-walled carbon nanotube electrode derivatized with the bifunctional linker cyclooctynyloxyethyl 1-pyrenebutyrate [123]. A remarkably high direct electron transfer efficiency and a stable current that deteriorated only around 14% following 8 days of solution-phase incubation at ambient temperature were achieved with the electrode containing the click-incorporated Glu47AzF variant of SLAC.

##### Inverse-Electron-Demand Diels-Alder Reactions (IEDDA)

The inverse-electron-demand Diels-Alder (IEDDA) reaction involves the cycloaddition of an electron-rich dienophile with an electron-poor diene (Scheme 15C). Cycloaddition of tetrazines with strained *trans*-cyclooctenes does not require a catalyst, shows a higher tunable reaction rate compared to 3+2 cycloadditions, and can be performed in different buffers, showing high biocompatibility. Human carbonic anhydrase II was genetically engineered with a tetrazine containing unnatural amino acid (Tet2.0) at three different positions (186, 233, 20) and immobilized via IEDDA onto *trans*-cyclooctene modified surfaces [124]. This immobilization strategy allowed the enzyme load to be controlled by modulating the amount of protein introduced into the system also at low protein concentration (nM). Moreover, the short reaction time (minutes) required for the immobilization procedure minimizes protein denaturation and nonspecific adsorption, allowing immobilized surfaces to be prepared with exceptional levels of homogeneity.

##### Glaser-Hay

The Glaser-Hay bioconjugation between terminal alkenes allows a highly stable and rigid linear C-C bond to be obtained under mild conditions (Scheme 15D) [125]. The hyperthermophilic carboxylesterase P1 from *Sulfolobus solfataricus* (SSo EST1) was immobilized onto an epoxy-activated Sepharose resin derivatized with propargyl alcohol via Glaser–Hay reaction [126]. Four tyrosine residues were mutated into the tyrosine derivative p-propargyloxyphenylalanine (pPrF) (Tyr90 Tyr116, Tyr191, and Tyr214). Incorporation of pPrF at sites 90 and 116 resulted in similar or slightly lower protein expression yields than wild-type; however, incorporation at residues 191 and 214 resulted in significantly decreased yields. The SSo EST1-116 mutant maintained comparable activity to the wild-type protein, suggesting minimal perturbations caused by pPrF, whereas the SSo EST1-214 mutant shows significantly reduced activity. The immobilized enzyme exhibited high performance in organic solvents, recyclability, and stability at room temperature for over 2 years.

## 4. Evaluation of Immobilization Outcome

Immobilization, considered the attachment of an enzyme on or into a porous solid carrier, is a relatively simple process. However, many factors can affect the expressed activity of the final enzyme derivative: enzyme distortion, mass transfer limitations, steric hindrance, enzyme leaching, carrier with low loading capacity, physical features of the carrier, etc. All these maters should be considered at the end of the immobilization procedure in order to fully understand the effects of the immobilization on enzyme activity. The three parameters that should be provided to evaluate the success of enzyme immobilization are the immobilization yield, the immobilization efficiency, and the activity recovery. These parameters allow the enzyme activity to be evaluated during the whole immobilization process.

The immobilization yield is used to describe the percentage of enzyme activity that has been immobilized onto the carrier. This parameter is calculated by dividing the residual activity found in the supernatant at the end of the immobilization procedure by the activity found initially in the supernatant before the addition of the solid carrier.

Immobilization yield (%) = (Units_t0_ − Units_endpoint_)/(Units_t0_) × 100

The immobilized activity at the endpoint is determined by measuring the total residual enzyme activity that remains in the supernatant at the endpoint of the immobilization and by subtracting this activity from the total initial activity. In order to be sure that the decrease of activity observed in the supernatant at the end of the immobilization is due to the effective binding of the enzyme to the carrier, a parallel stability experiment should be carried out in order to evaluate the possible deactivation of the soluble enzyme in the immobilization conditions. The evaluation of protein concentration during the immobilization can also be used to determine the immobilization yield. However, the disappearance of the protein from the supernatant sometimes could be misleading, especially when a crude protein mixture is used because the different proteins present in the mixture can have different immobilization yields. Generally, both protein concentration and enzyme activity have to be monitored during an immobilization process in order to be able to rule out any deactivation of the soluble enzyme and to determine the protein loading of the immobilized biocatalyst [1,19].

The immobilization efficiency describes the percentage of enzyme activity that was bound into the final derivative. These parameters take into account the activity of the final immobilized derivative and the immobilization yield [1,19].

Efficiency (%) = (observed activity (U/g))/(immobilized activity (U/g)) × 100

Finally, the activity recovery describes the overall success of an immobilization protocol. It correlates the initially loaded activity and the final activity observed in the immobilized biocatalyst [1,19].

Activity recovery (%) = (observed activity (U/g))/(loaded activity (U/g)) × 100

These parameters should be reported in all papers dealing with enzyme immobilization in order to allow the reader to fully understand what is going on during the immobilization process as well as the properties of the final immobilized derivative obtained.

## 5. Conclusions

Protein immobilization represents a versatile and promising solution to address the challenges associated with enzyme applications in industrial processes.

This comprehensive review highlights the numerous advantages offered by immobilization techniques, including enhanced enzyme stability, simplified recovery and reuse, and significant reductions in downstream processing and operational costs. These benefits make immobilization a cornerstone for developing sustainable and efficient biocatalytic processes.

Classical non-specific immobilization methods, such as adsorption, ionic binding, and entrapment, are widely utilized due to their simplicity, cost-effectiveness, and high enzyme loading capacities. However, these approaches often lack precise control over enzyme orientation, which can negatively impact catalytic efficiency and stability. Covalent immobilization methods provide stronger enzyme-support interactions and improved stability, but they require careful optimization to minimize activity loss caused by conformational changes during the process.

Oriented immobilization methods, leveraging advanced protein engineering techniques, represent a significant step forward in maximizing enzyme activity. By introducing specific functional tags or unique amino acid residues, these methods ensure the optimal orientation of enzymes on supports, improving substrate accessibility and catalytic efficiency. Such approaches are particularly valuable for applications where enzyme activity and selectivity are critical, such as pharmaceutical synthesis or fine chemical production.

Despite the clear advantages, challenges remain. Immobilization can lead to mass transfer limitations, reduced activity with insoluble substrates, or enzyme denaturation if protocols are not carefully designed. Hybrid approaches that combine protein engineering and immobilization offer a promising solution to overcome these limitations, enabling the development of tailored biocatalysts that exhibit both high stability and activity.

## Figures and Tables

**Table 1:** Most common reactive groups naturally present on enzyme surface used for immobilization through covalent bond formation.

Reactive Group	Structure	Amino Acid
Primary amine	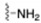	*N*-terminus ε-amino groups of Lysine
Carboxylic acid	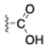	*C*-terminus Glutamic acid Aspartic acid
Thiol	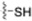	Cysteine
Imidazole	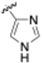	Histidine

**Table 2:** Most common functional groups present on carriers and used for covalent bonds with enzymes.

Functional Group	Structure	Binding	Reactive Group of the Enzyme
Epoxy	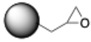	Nucleophilic attach and epoxy ring opening	Nucleophilic groups (-NH_2_ and -SH)
Amino	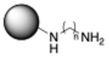	Activation with a dialdehyde; Schiff base formation	Primary amines (terminal-NH_2_ and Lys side chains)
Hydroxy	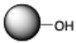	Activation with a dialdehyde; Schiff base formation	Primary amines (terminal-NH_2_ and Lys side chains)
		Activation with DVS; C-X bond formation	Imidazole, thiol and primary amines (depending on the pH)
		Activation with CnBr; imidocarbonate bond formation	Primary amines (terminal-NH_2_ at mild pH)
		Activation with CDI; carbamate bond formation	Primary amines (terminal-NH_2_ and Lys side chains)
		Activation with epoxy groups (GLYMO); Nucleophilic attach and epoxy ring opening	Nucleophilic groups (-NH_2_ and -SH)
		Activation with thiol groups; thiol exchange (S-S bond)	-SH

Legend: DVS = divinyl sulfone; CNBr = cyanogen bromide; CDI = *N*,*N*′-carbonyldiimidazole; GLYMO = (3-glycidyloxypropyl)trimethoxysilane.

## Data Availability

No new data were created or analyzed in this study.
